# Sleep problems in opioid dependent patients maintained on buprenorphine

**DOI:** 10.1192/j.eurpsy.2021.1486

**Published:** 2021-08-13

**Authors:** R. Tripathi, R. Rao, A. Dhawan, R. Jain

**Affiliations:** 1 Psychiatry, All India Institute of Medical Sciences, Gorakhpur, Gorakhpur, India; 2 Psychiatry, All India Institute of Medical Sciences, New Delhi, Gorakhpur, India

**Keywords:** opioid dependence, buprenorphine, sleep, sleep problems

## Abstract

**Introduction:**

Opioid dependent individuals frequently complain of sleep problems in withdrawal and during abstinence.

**Objectives:**

The objectives were to assess the subjective sleep parameters among buprenorphine-maintained opioid-dependent patients and to correlate it with socio-demographics, concomitant drug use and treatment related variables

**Methods:**

Using a cross-sectional study design, 106 hundred six opioid-dependent patients maintained on buprenorphine for at least six months and on same dose in past month were interviewed. Sleep was assessed by Pittsburgh sleep quality index (PSQI) and Epworth sleepiness scale. Association between subjective sleep parameters, socio-demographics, concomitant drug use and treatment related variables was also studied.

**Results:**

All participants were males. Their mean age was 41.1 years (SD:14.3). The mean duration of illicit opioid use was 10 years (IQR: 5,22). About 63.2% (n=67) had PSQI scores more than 5 denoting sleep problem. The scores obtained in Epworth Sleeping Scale were in normal range. Mean subjective total sleep time of the sample was 403.5 (SD 94.8) minutes and median sleep latency was 35 (IQR 18.8, 62.5) minutes. Subjective total sleep time was significantly higher in participants who had use tobacco in the past three months (p value=0.03) and who were in moderate ASSIST risk category (p value=0.04). Subjective sleep latency was significantly higher (p value=0.04) in participants who had used opioids in last three months. It was observed that age was a significant predictor of subjective total sleep time and OST compliance was a significant predictor of sleep latency.

**Conclusions:**

A sizeable proportion of opioid dependent patients on buprenorphine have sleep problems

